# A New Human Leukocyte Antigen Typing Algorithm Combined With Currently Available Genotyping Tools Based on Next-Generation Sequencing Data and Guidelines to Select the Most Likely Human Leukocyte Antigen Genotype

**DOI:** 10.3389/fimmu.2021.688183

**Published:** 2021-10-01

**Authors:** Miseon Lee, Jeong-Han Seo, Sungjae Song, In Hye Song, Su Yeon Kim, Young-Ae Kim, Gyungyub Gong, Jeong Eun Kim, Hee Jin Lee

**Affiliations:** ^1^ Department of Hospital Pathology, Seoul St. Mary’s Hospital, College of Medicine, The Catholic University of Korea, Seoul, South Korea; ^2^ Department of Biomedical Sciences, Asan Medical Institute of Convergence Science and Technology, University of Ulsan College of Medicine, Asan Medical Center, Seoul, South Korea; ^3^ NeogenTC Corp, Seoul, South Korea; ^4^ University of Ulsan College of Medicine, Seoul, South Korea; ^5^ Department of Pathology, University of Ulsan College of Medicine, Asan Medical Center, Seoul, South Korea; ^6^ Department of Oncology, University of Ulsan College of Medicine, Asan Medical Center, Seoul, South Korea

**Keywords:** human leukocyte antigen (HLA), HLA genotype, next-generation sequencing data (NGS), HLA typing algorithm, immunotherapy, neoantigen

## Abstract

**Background:**

High-precision human leukocyte antigen (HLA) genotyping is crucial for anti-cancer immunotherapy, but existing tools predicting HLA genotypes using next-generation sequencing (NGS) data are insufficiently accurate.

**Materials and Methods:**

We compared availability, accuracy, correction score, and complementary ratio of eight HLA genotyping tools (OptiType, HLA-HD, PHLAT, seq2HLA, arcasHLA, HLAscan, HLA*LA, and Kourami) using 1,005 cases from the 1000 Genomes Project data. We created a new HLA-genotyping algorithm combining tools based on the precision and the accuracy of tools’ combinations. Then, we assessed the new algorithm’s performance in 39 in-house samples with normal whole-exome sequencing (WES) data and polymerase chain reaction–sequencing-based typing (PCR-SBT) results.

**Results:**

Regardless of the type of tool, the calls presented by more than six tools concordantly showed high accuracy and precision. The accuracy of the group with at least six concordant calls was 100% (97/97) in HLA-A, 98.2% (112/114) in HLA-B, 97.3% (142/146) in HLA-C. The precision of the group with at least six concordant calls was over 98% in HLA-ABC. We additionally calculated the accuracy of the combination tools considering the complementary ratio of each tool and the accuracy of each tool, and the accuracy was over 98% in all groups with six or more concordant calls. We created a new algorithm that matches the above results. It was to select the HLA type if more than six out of eight tools presented a matched type. Otherwise, determine the HLA type experimentally through PCR-SBT. When we applied the new algorithm to 39 in-house cases, there were more than six matching calls in all HLA-A, B, and C, and the accuracy of these concordant calls was 100%.

**Conclusions:**

HLA genotyping accuracy using NGS data could be increased by combining the current HLA genotyping tools. This new algorithm could also be useful for preliminary screening to decide whether to perform an additional PCR-based experimental method instead of using tools with NGS data.

## Introduction

The major histocompatibility complex (MHC) is located on chromosome 6p21.3 in humans, occupying a continuous 3.6 Mb segment of the human genome (1). The MHC has three distinct loci: classes I, II, and III. Class I and II loci belong to the subgroup associated with HLA genes. HLA genes encode cell-surface antigen-presenting proteins, which play a central role in discriminating self and non-self. Class I (HLA-A, HLA-B, and HLA-C) and II genes (HLA-DP, HLA-DQ, and HLA-DR) are responsible for presenting processed antigens to cytotoxic T cells and helper T cells, respectively. The HLA gene cluster is highly polymorphic and co-dominantly inherited from a parent. Thus, an individual has a distinct HLA allele combination called an HLA haplotype (an entire set of HLA-A, -B, -C, -DP, -DQ, and -DR). As of January 2021, over 29,000 HLA allele variants have been reported according to the IPD-IMGT (ImMunoGeneTics)/HLA reference database (release 3.43) (2).

HLA genes are associated with graft/transplant rejection, combating infectious disease, autoimmunity, and cancer, so they play important roles in a clinical context. Successful transplantation of solid organ/allogeneic stem cells is highly reliant on accurate prediction of the HLA genotype in order to find a suitable donor with concordant HLA alleles. Thus, HLA typing technologies have evolved from simple serologic methods to molecular analysis and next-generation sequencing (NGS) technology. The serologic methods use an antigen/antibody response and have limitations regarding the dependence on cellular viability and low resolution (two-digit typing). These approaches have been superseded by polymerase chain reaction (PCR)-based molecular methods focusing on deciphering the sequences of exons 2 and 3 (~540 bp) of class I and exon 2 (~270 bp) of class II, which are responsible for encoding the peptides of antigen-binding clefts and polymorphisms of HLA gene. Commonly used PCR-based molecular approaches include use of a sequence-specific oligonucleotide probe (SSOP) (3), sequence-specific primers, and SBT. Compared with other molecular techniques, SBT provides a higher resolution of HLA genotyping by sequencing polymorphic regions using PCR and has become a standard method based on PCR. However, PCR-SBT is time-consuming, labor-intensive, expensive, and produces ambiguous data from both heterozygous alleles together in a single reaction (4).

NGS technology can replace these methods by enabling high-throughput sequencing with high resolution, less ambiguity, and reduced labor, time, and cost. However, HLA genotyping by NGS still fails to meet expectations due to focusing on numerous similar genes and pseudogenes, genetic complexity, difficulty in finding a reference genome, and the presence of segmental duplications. Various HLA genotype predicting tools based on NGS data have been developed to resolve these issues as follows: HLA_MINER_, ATHLATES (5), HLA_REPORTER_ (6), OptiType (7), HLA-HD (8), PHLAT, seq2HLA (9), arcasHLA (10), HLAscan (11), HLA*LA (12), Kourami (13), HLA-VBSeq (14), HLA-VBSeq.V2 (15), PolySolver, HLAforest, xHLA (16), and HLAProfiler (17). These tools are based on broadly two methods, including alignment-based and assembly-based methods. The alignment-based methods align the sequencing read obtained from whole-genome sequencing (WGS), WES, whole-transcriptome sequencing, or amplicon datasets to the reference database and then select the best matching allele in the reference database alignment statistics, including the extent of exon coverage and the number of reads covering exons (18). The assembly-based method assembles the reads to contigs and map these reads into the reference database (19).

With recent developments of medical technology, anti-cancer immunotherapy based on neoantigens is gradually attracting attention as alternative personalized medicine in cancer patients, given the limited efficacy of current anti-cancer treatments. Neoantigens are mutant proteins generated from somatic mutations of tumors. Vaccines attacking tumors with neoantigens can be produced and used as therapeutic agents. T cells with T-cell receptors (TCRs) capable of targeting neoantigens can be selected and cultured *in vitro*. TCR-engineered T cells can be manufactured and used as therapeutic agents. However, these therapies’ success relies on various factors, and one of the significant factors is to enable high-precision HLA genotyping. Even if patients have the same mutation, the corresponding mutant protein may or may not be presented as a neoantigen, depending on each patient’s HLA genotype. If a peptide derived from the mutant protein cannot be bound to HLA, it cannot be presented as a neoantigen. Therefore, it is crucial to accurately identify HLA genotype to determine whether it can be a neoantigen and be used in anti-cancer immunotherapy. Errors in HLA typing must be avoided in these treatments, but current tools predicting HLA genotypes using NGS data are insufficiently accurate.

This study compared the accuracy of HLA genotyping of reported HLA genotyping algorithms using NGS data of the 1000 Genomes Project (20). We developed a new algorithm with higher accuracy by combining existing tools, suggesting guidelines to select the most likely HLA genotype.

## Materials and Methods

### NGS Dataset From the 1000 Genomes Project

To investigate the predictive performance of the algorithm tools, we first obtained WES data based on NGS from 1,005 individuals from the 1000 Genomes Project (https://www.1000genomes.org). We also obtained additional HLA genotype information for HLA class I genes (*HLA-A, -B, -C*) analyzed using PCR-SBT considered as the gold standard for 1,005 individuals from the project (https://www.internationalgenome.org/category/hla/). We got the HLA genotype at four-digit resolution and used it as an HLA genotype reference.

Sequencing data of the 1,005 samples were obtained from Illumina’s platforms (HiSeq 2000, 825 cases; Genome Analyzer II, 180 cases). Paired-end read length was 76 bp (272 cases), 90 bp (295 cases), 100 bp (186 cases), and 101 bp (252 cases).

### Evaluation of HLA Genotyping Performance of Currently Available Algorithms

**Figure 1:**
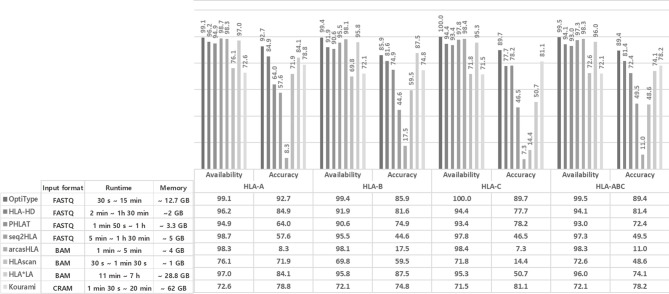
Performance comparison of eight tools using the 1000 Genomes Project data. The availability, accuracy of the eight tools in each of HLA-A, B, and C. Availability refers to how many of the eight tools present a specific call, and accuracy refers to how identical the call is comparing with 1000 Genomes Project data. Runtime and memory of the eight tools in each case. The different runtime for each tool is checked from the minimum time to the maximum time, and the maximum memory consumed is displayed.

Using WES data on 1,005 individuals based on NGS from the 1000 Genomes Project, HLA genotyping for *HLA-A, -B*, and *-C* was performed with eight available algorithms, namely, OptiType, HLA-HD, PHLAT, seq2HLA, arcasHLA, HLAscan, HLA*LA, and Kourami, in accordance with the instructions of each tool (**Table 1**). As input data, FASTQ files were used for OptiType, HLA-HD, PHLAT, and seq2HLA, BAM files were used for HLAscan, arcasHLA, and HLA*LA, and CRAM files were used for Kourami (**Figure 1**).

**Table 1:** Overview of tools.

Program	URL	Read type	MHC class	Resolution	Input format
OptiType	https://github.com/FRED-2/OptiType	WGS/WES/RNA-Seq	Class I	2 fields	FASTQ
HLA-HD	https://www.genome.med.kyoto-u.ac.jp/HLA-HD/	WGS/WES/RNA-Seq	Class I and II	3 fields	FASTQ
PHLAT	https://sites.google.com/site/projectphlat/Downloads	WES/RNA-Seq	Class I and II	3 fields	FASTQ
seq2HLA	https://github.com/TRON-Bioinformatics/seq2HLA	RNA-Seq	Class I and II	2 fields	FASTQ
arcasHLA	https://github.com/RabadanLab/arcasHLA	RNA-Seq	Class I and II	3 fields	BAM
HLAscan	http://genomekorea.com/display/tools/HLAscan	WGS/WES	Class I and II	4 fields	FASTQ/BAM
HLA*LA	https://github.com/DiltheyLab/HLA*LA	WGS, WES	Class I and II	3 fields	BAM/CRAM
Kourami	https://github.com/Kingsford-Group/Kourami	WGS	Class I and II	3 fields	BAM/CRAM

We defined the available call when the tool predicts the type as one prediction. Thus, we classified the unavailable call if the tool does not present predictions or present multiple predictions such as 03:01/05/06/50 in any one of the two alleles. We evaluate the availability by calculating the number of samples with both available calls in two alleles in each type.


$$\text{Availability}=\frac{\mathrm{Samples with both available calls in two alleles}}{\text{Total samples}}*100$$

The generated calls for HLA genotyping were then compared to the HLA haplotype reference of the 1000 Genomes Project and analyzed for accuracy at the four-digit resolution. We evaluate the accuracy for each of *HLA-A*, *-B*, and *-C* by calculating the number of samples with both concordant alleles with reference from the 1000 Genomes Project in each tool.


$$\text{Accuracy}=\frac{\text{Samples with both matched calls to reference in two alleles}}{\text{Samples with both available calls in two allels}}*100$$

### The Concordance of Eight Tools’ Call and the Accuracy of the Concordant Call

Using the R project (R version 4.1.0, Vienna, Austria) and UpSetR package (21), we calculated and visualized eight tool’s call and concordance. We evaluated the number of matches among the calls presented by eight tools and calculated the accuracy of the matched calls.

### The F1 Score of the Concordant Call According to the Number of Concordant Calls

According to the number of concordant calls presented from eight tools, we calculated the number of the concordant calls that match (“true calls”) or do not match (“false calls”) to the reference. Using these results, we calculated the F1 score. We obtained the F1 score in the following way, where “true positive” refers to “true call”, and “false positive” corresponds to “false call”. “False-negative” means the concordant call corresponding to the reference in the rest of the group.


$$\begin{array}{l} \text{F}1\text{ score}=2*\frac{\text{precision}*\text{recall}}{\text{precision}+\text{recall}}\times 100,\text{ precision}=\frac{\text{TP}}{\text{TP}+\text{FP}},\text{ recall}=\frac{\text{TP}}{\text{TP}+\text{FP}}\\ \text{ TP};\mathrm{true positive},\text{FP};\mathrm{false positive},\text{FN}:\text{false negative} \end{array}$$

### Correction Score, Complementary Score, and Complementary Ratio

When the call of one tool presented a different call from the reference, we compared the correspondence of the calls of the other tools with the reference and calculated how much the call of the other tools corrects when the call of one tool is wrong. Assuming there were tools A and B, we calculated how much B’s call is true when A’s call was wrong and how much A’s call is correct when B’s call was incorrect. Then, we defined each value as the correction score of B to A and A to B. We defined the average of these two values as the complementary score between A and B. We also calculated the complementary ratio by additionally considering the accuracy of the tool with the correction score.


Correction score of B to A=The number of B succeeded to call the correct answer when A failed to guessThe  number of A failed to call the correct answerComplementary score between A and B=(Correction score of B to A)+(Correction score of A to B)2Complementary ratio of B to A=(1-correction score of B to A)(1−B's accuracy)

### In-House Sample Preparation

We included an additional in-house dataset comprising 39 samples from different cancer patients (5 breast cancers, 15 cholangiocarcinomas, 4 colorectal cancers, 5 lung cancers, 5 stomach cancers, and 5 sarcomas). All samples were obtained under Institutional Review Board approval with written informed consent.

Using buffy coat DNA from peripheral blood or formalin-fixed paraffin-embedded normal tissue, we conducted PCR-SBT and HLA typing. Additional normal WES data were obtained using 101-bp paired-end reads on Illumina HiSeq 2500 using SureSelectXT Library Prep Kit.

## Results

### Performance of Eight Currently Available Algorithms for HLA Genotyping

In many cases of 1,005 samples, HLA genotypes obtained from 1000 Genomes using PCR-SBT were multiple types or were not available. The number of cases obtained the HLA genotype as one type was 234 for HLA-A, 308 for HLA-B, and 309 for HLA-C. Using this data, we calculated the availability and accuracy of eight HLA typing tools (OptiType, HLA-HD, PHLAT, HLAscan, HLA*LA, seq2HLA, arcasHLA, and Kourami) in the typing of each of HLA-A, HLA-B, HLA-C, and HLA-ABC at the four-digit resolution (**Figure 1**).

The availability of HLA genotyping was the highest in the OptiType tool for all HLA-A, C, and B types, and the values were 99.1% for HLA-A, 99.4% for HLA-B, and 100% for HLA-C. In HLA-A and HLA-C, Kourami showed the lowest availability, and the values were 72.6% and 72.5% in each. In HLA B type, HLAscan showed the lowest availability, and the value was 69.8%.

For accuracy, OptiType showed the highest accuracy in HLA-A and HLA-C, and the values were 92.7% and 89.7%, respectively. In HLA-B, HLA*LA showed the highest accuracy, and the value was 87.5%. arcasHLA showed the lowest accuracy in all of HLA-A (8.4%), B (17.5%), and C (7.3%).

The runtime in each case required varies depending on the input format, read length, etc. OptiTpye (30 s ~ 15 min) and HLAscan (30 s ~ 1 min 30 s) took a relatively short time, and HLA*LA (11 min ~ 7 h) took a longer runtime than other tools.

HLAscan (~ 1 GB) required a relatively small amount of memory among tools, and Kourami required a relatively large amount of memory (~63 GB).

### The Concordance of the Eight Tools’ Calls and the High Accuracy in the Group With at Least Six Concordant Calls

**Figure 2:**
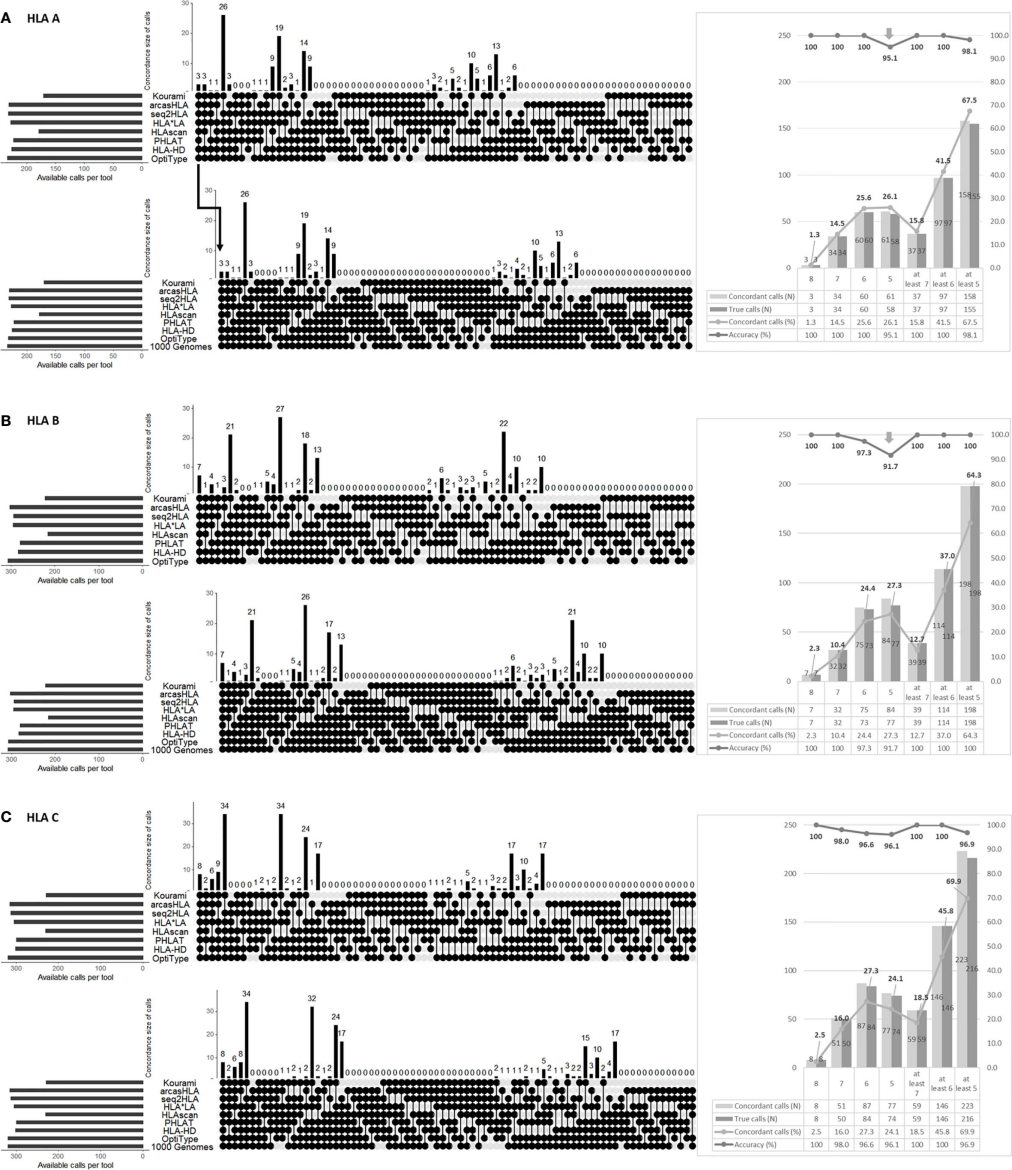
Performance analysis of HLA genotyping by combining the eight tools in HLA-A **(A)**, HLA-B **(B)**, HLA-C **(C)**. The upper part in each **(A–C)** shows the combinations of tools presenting the concordant calls. The round dots indicate the tools included in the combination. The number is the cases of each combination. The lower part in each **(A–C)** shows the accuracy of each combination. Each combination’s number in the lower part means that the concordant calls matched with the 1000 Genome Project data (i.e., “1000 Genome” points are additionally included in the combination). Therefore, the accuracy is the number of lower parts (the number of concordant calls matched with 1000 Genome Project data) divided by the number of upper parts (the number of concordant calls). The right part of each of **(A–C)** shows the number and percentage of concordant calls according to the number of matched calls regardless of the tool type and the number of true calls among these concordant calls and their percentage.

We calculated the concordance of the eight tool’s calls and the accuracy of the concordant call (**Figure 2**).

In HLA-A, at least six tools presented the same call in 41.5% (97/234), and the accuracy of the concordant calls was 100% (97/97). However, the accuracy of the five concordant calls was fell to 95.1% (58/61).

In HLA-B, at least seven tools presented the same call in 12.7% (39/308), and the accuracy of the concordant calls was 100% (39/39). The accuracy of six concordant calls was 97.3% (73/75), and the accuracy of five concordant calls dropped to 91.7% (77/84).

In HLA-C, only the accuracy of the eight concordant calls was 100% (8/8). The accuracy of the seven concordant call was 98.0% (50/51). The accuracy of the six and five concordant calls decreased to 96.6% (84/87) and 96.1% (74/77).

In all types, the accuracy increases as the number of concordant calls increases.

### High Precision in the Group With Over Six Concordant Calls

**Figure 3:**
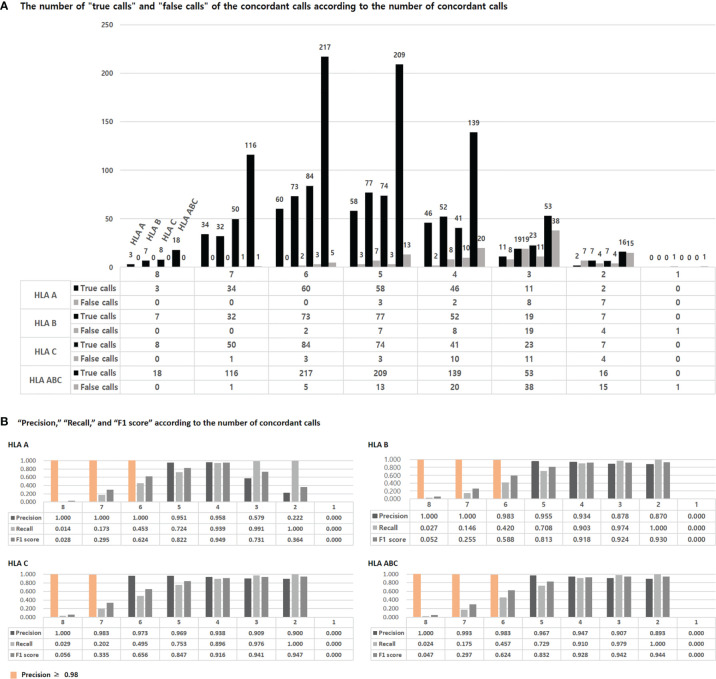
The number of “true calls” and “false call” of concordant calls according to the number of the concordant calls **(A)**. The “true calls” means that the number of calls that match the HLA type of the 1000 Genome Project data among the concordant calls presented by eight tools. The “false calls” means that the number of calls that do not match the HLA type of the 1000 Genome Project data among the concordant calls presented by eight tools. “Precision”, “Recall”, and “F1 score” according to the number of concordant calls **(B)**. Precision and recall are calculated using “true calls” and “false calls”, and F1 scores are obtained using these two. The detailed formula is given in the text.

We calculated the “true call” and “false calls” of the concordant calls according to the number of concordant calls (**Figure 3A**). In HLA-ABC, the number of false calls was small, less than 10, in eight, seven, and six concordant calls, but the number of false calls was 13 in five concordant calls.

We calculated precision, recall, and F1 scores using the “true calls” and “false calls” of the concordant calls according to the number of concordant calls (**Figure 3B**). The precision was 1 in over six concordant calls of HLA-A, over seven concordant calls of HLA-B, and eight concordant calls of HLA-C. In HLA-A, B, and ABC, the precision was over 0.98 in over six concordant calls. In HLA-C, the precision was over 0.98 in over seven concordant calls. The F1 score was highest in four concordant calls for HLA-A, two concordant calls for HLA-B, C, and ABC.

### High Correction Score of OptiType, HLA-HD, PHLAT, and HLA*LA and High Complementary Ratio of OptiType, HLA-HD, and PHLAT

**Figure 4:**
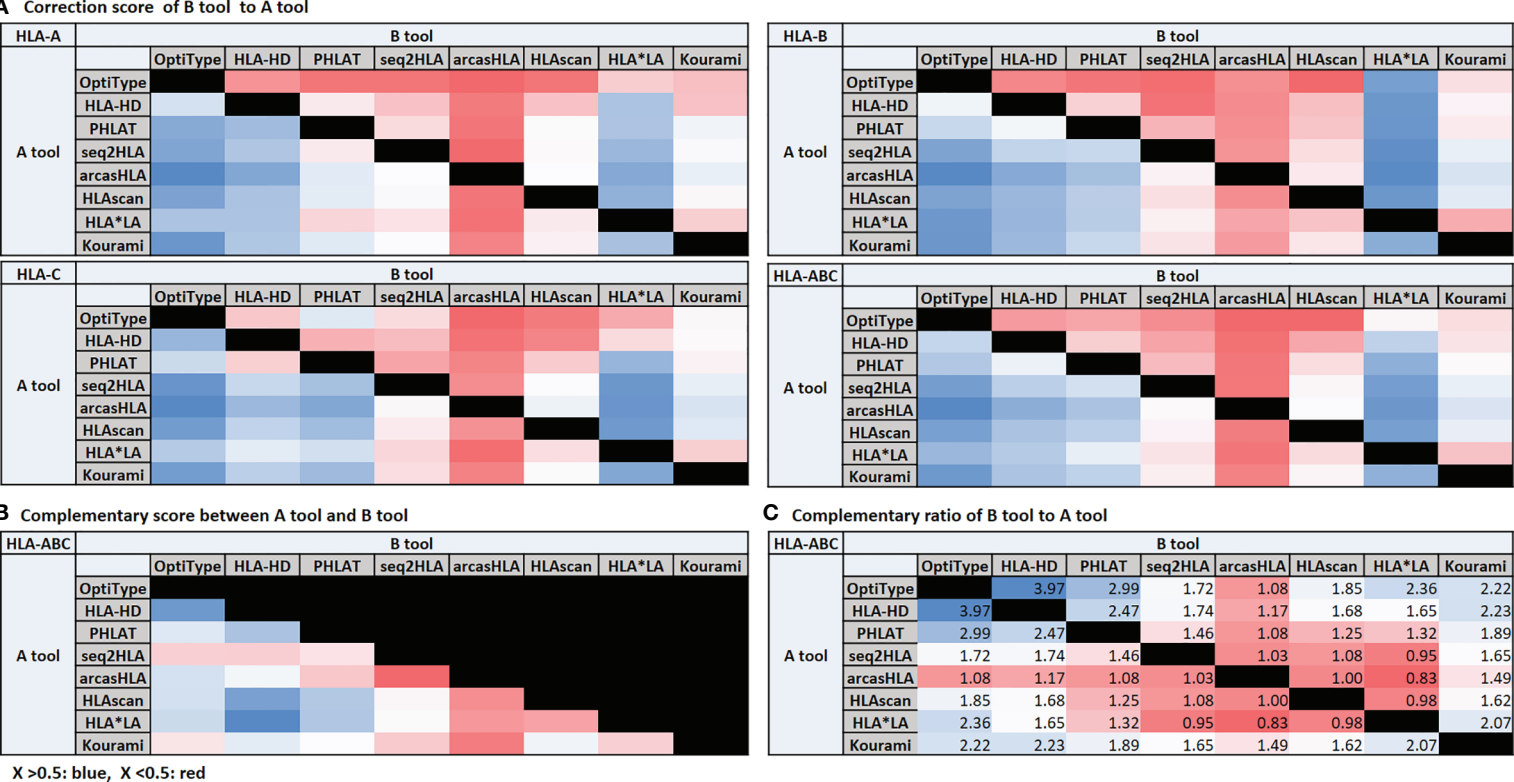
Correction score **(A)** and complementary score **(B)**. The correction score is calculated by measuring how much the calls of other tools match the reference when a call of one tool is wrong. Assuming that there are tools **(A, B)**, the correction score of **(B, A)** is the degree to which the call of **(B)** is correct when the call of **(A)** is incorrect. The degree to which the call of **(A)** is correct when the call of **(B)** is incorrect is the correction score of **(A, B)**. The average of these two scores is the complementary score between **(A, B)**. The complementary ratio **(C)**. The complementary ratio of **(B, A)** is obtained by additionally considering the accuracy of the tool with the correction score.

We organized the correction scores (**Figure 4A**), the complementary score (**Figure 4B**), and the complementary ratio (**Figure 4C**) of eight tools. OptiType, HLA-HD, PHLAT, and HLA*LA showed high correction scores. The complementary scores between OptiType and HLA-HD, HLA-HD and PHLAT, HLA-HD and HLAscan, HLA-HD and HLA*LA were high. OptiType, HLA-HD, and PHLAT showed high complementary ratios. We additionally calculated the accuracy of the combination tools considering the complementary ratio of each tool and the accuracy of each tool, and the accuracy was over 98% in all groups with six or more concordant calls.

### Development of a New Algorithm by Combining Currently Available Tools and the Workflow of Combined Algorithm to Determine HLA Genotype

Considering the results, we can hypothesis that we could develop a new algorithm by combining currently available algorithms. We created the algorithm based on the group with high accuracy and precision in the 1000 Genomes Project data.

Regardless of the type of tool among the eight tools, in the groups with six or more tools present the same call, the group showed high accuracy with high precision.

Based on the above results, we developed guidelines to select an optimized HLA type.

1. Regardless of the type of tool among the eight tools, if only six or more tools present the same call, choose the call as HLA genotype.
2. In the remaining cases in which No. 1 do not apply, determine the HLA type experimentally through PCR-SBT.

Using Python (version 3.8.3), we created a calling program (https://github.com/k1k2k311/HLA-combined) that contained our new algorithm (saved as “Simple calling.py”). This program would inform you of the specific HLA type if the type matched with the new algorithm. Otherwise, it would announce you “Recommend PCR-SBT”. We also developed a program (saved as “custom calling.py”) that would inform us which combinations of concordant calls exceed a certain accuracy threshold. The accuracy here refered to the accuracy considering the complementary ratio of each tool. For example, suppose we would like to know whether the concordant call of the specific tools’ combination exceeds the accuracy of 98%. In that case, we could enter the threshold as 98% and insert the combinations’ calls. Then, the program would inform us of the concordant calls’ type if the concordant calls’ accuracy over 98%. This program was generated using each tool’s accuracy (saved as “model_acc.csv”) and complementary ratio (saved as “cor_score.csv”). We analyzed using this program whether the accuracy exceeds 98% in the six or more concordant calls regardless of the tool type, and all of them exceeded 98%.

### High Performance of the New Algorithm in In-House Sample

**Figure 5:**
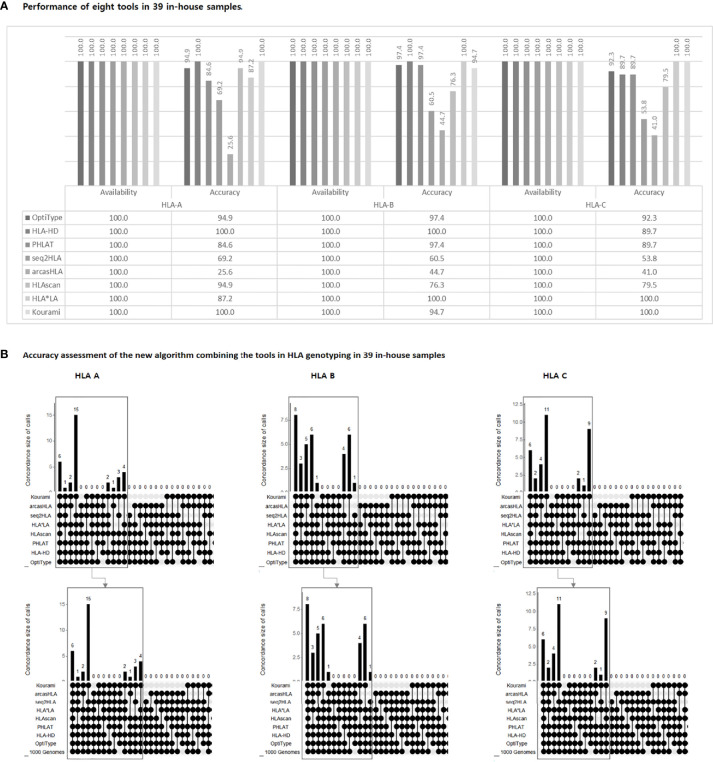
Performance of eight tools in 39 in-house samples **(A)**. The availability and accuracy of each of the eight tools in each of HLA-A, B, and C Accuracy assessment of the new algorithm combining the tools in HLA genotyping in 39 in-house samples **(B)**. The upper part in each HLA-A, B, and C shows the combinations of tools presenting the concordant calls. The round dots indicate the tools included in the combination. The number is the cases of each combination. The lower part in each HLA-A, B, and C shows the accuracy of each combination. Each combination’s number means that the concordant calls matched with the 1000 Genome Project data (i.e., “1000 Genome” points are additionally included in the combination). Therefore, the accuracy is the number of lower parts (the number of concordant calls matched with 1000 Genome Project data) divided by the number of upper parts (the number of concordant calls). The rectangle shows the case of 6 or more concordant calls (corresponding to the new algorithm), and when matched with the following, 100% accuracy is demonstrated in these cases.

To assess the new algorithm’s performance, we included 39 in-house samples. All 39 cases in HLA-A and HLA-C and 38 cases in HLA-B had available allele calls obtained experimentally by PCR-SBT. All cases obtained available calls from all of eight tools. We calculated the accuracy of HLA-A, HLA-B, and HLA-C for each of the eight tools. Some tools showed 100% accuracy, but the tools were different in HLA-A, HLA-B, and HLA-C, respectively, and none of the tools showed 100% accuracy in all three HLA-A, B, and C types at the same time (**Figure 5A**).

The groups with at least six tools presented the same call were 34/39 (87.2%) in HLA-A, 34/38 (89.5%) in HLA-B, and 35/39 (89.7%) in HLA-C, and the accuracy of the concordant calls was 100% in all type (**Figure 5B**).

## Discussion

The rapid development of NGS technology has significantly influenced medical genomics by providing sequencing data from patient samples. Sequencing projects such as the 1000 Genomes Project, UK10K Project (http://www.uk10k.org), and NHLBI GO Exome Sequencing Project (https://esp.gs.washington.edu) have provided vast WGS or WES datasets, which improve our understanding of the causes of disease and variations in the human genome. NGS tests are currently actively being conducted by focusing on targeted genes related to cancer and are used for patient treatment, leading to the increased availability of HLA typing using NGS data. Computational software tools to predict HLA type based on NGS data have increasingly been reported (6–15). However, there is no standard method to provide the perfect assignment for all HLA types given the difficulty of overcoming polymorphisms and the complexity of HLA, the similarity between different HLA allele sequences, and the limited stored reference databases of each HLA typing tool (18). All HLA typing tools have their own advantages and limitations, and no tool achieves the required accuracy for medical applications. Therefore, we attempted to create a new algorithm for accurate HLA typing using NGS data, using currently available HLA typing tools.

We developed a new algorithm by combining currently available HLA typing software, using 1,005 WES data of the 1000 Genomes Project data. However, only 234 samples in HLA-A, 308 samples in HLA-B, and 319 samples in HLA-C had available HLA genotyping results obtained from PCR-SBT. The 1000 Genomes Project data were obtained more than 10 years ago, considering, the quality of the data reference is limitation of our study.

This algorithm was created based on the group with high accuracy and. We chose precision instead of the F1 score because in the medical field, knowing whether a specific HLA genotype obtained as a result of the analysis is a true value is more important than knowing as many types as possible that are likely to be HLA genotypes. In other words, reducing false-positive values is much more critical than lowering false negative values. However, the low F11 score in the group with large concordant calls showing high precision and the high F1 score in the small concordant calls group was a problem not to be ignored. We speculated that these values ​​were derived because the number of samples was too small and the depth of the sequence was too small.

This algorithm is to select the type presented concordantly from the different software programs. To assess the performance of this new algorithm, we included 39 additional in-house samples, and the accuracy was 100% in all of HLA-A, HLA-B, and HLA-C. In the remaining cases in which the new algorithm with high accuracy and precision was not satisfied, we think that obtaining the HLA type experimentally through PCR-SBT is more appropriate than using NGS data-based HLA typing tools. One of the reasons for low accuracy is that the case has a rare allele of the HLA gene that is not present in each tool’s reference (18, 22). Alternatively, it may be related to the 1000 Genomes Project data having sequencing depth that is insufficient for clinical HLA typing. Thus, the HLA genotyping performance of tools may not reflect the capability fully (19).

The new algorithm increased HLA genotyping accuracy compared with currently available HLA genotyping tools using NGS-based data. However, our study had a limitation in covering the gene region associated with HLA genotyping by using the 1000 Genome Project data, for which analysis is only at the four-digit resolution level and only exome information is contained (19). This may be related to the low accuracy of tools and the presence of many cases that do not satisfy the new HLA typing algorithm because some HLA typing tools need more information, rather than just exome information. HLA typing resolution is divided into four categories: two-digit resolution for allele group, four-digit resolution for specific HLA protein, six-digit resolution for specific HLA coding sequence, and eight-digit resolution for specific HLA genome sequence, including introns and untranslated regions (18). Alterations in introns and untranslated regions may lead to splicing or other defects and affect HLA gene expression. Thus, the determination of all regions, including the HLA genes and associated regulatory sequences, is necessary to obtain a sufficient understanding of HLA phenomena (18, 23). Therefore, to apply the new algorithm in a clinical context, research at a higher resolution than four-digit resolution is required. Full HLA gene sequencing, rather than sequencing of the coding sequence or partial exons, will help achieve eight-digit resolution and increase the detection of new or rare alleles for more clinical applications. Neither the 1000 Genomes Project data nor the in-house data were from the tumor tissue. For HLA type to be applied to tumor-associated immunotherapy, including vaccination and T-cell-mediated therapy, HLA typing analysis has to be performed in tumor tissue because somatic mutation associated with the HLA gene could occur in the tumor. We developed our new algorithm based on tools’ combinations with high precision and accuracy, but it was not 100%. However, for application in a clinical context, there is a need to increase the accuracy up to 100%. Some studies revealed that each HLA genotyping tool has a specific length with high accuracy (24). However, our study did not compare according to read length, which is also a limitation. Our study was analyzed using only the WES dataset. We tried to add the WGS dataset, but it failed because it took too long to download. We could obtain only 19 cases with both WES and WGS datasets. Although the number of samples was too small we found out that the WGS dataset’s accuracy was significantly lower than the WES dataset, and the degree was more severe in the low coverage than in the high coverage. Considering these results, we assumed that the depth of the WGS dataset had a significant effect. Therefore, additional data analysis considering depth was also necessary, but not doing so is also our limitation.

The new algorithm would increase the precision of HLA typing to the extent that it can be applied to clinical applications. These results suggest that the combination of the current algorithm tools might be helpful for predicting the accurate HLA genotypes using NGS-based data. It could also be a useful preliminary screening tool to determine whether to perform an additional PCR-based experimental method.

## Data Availability Statement

Our data has been deposited to the European Genome-phenome Archive and can be accessed *via* the following accession numbers: EGAD00001007733, EGAS00001005274.

## Ethics Statement

The studies involving human participants were reviewed and approved by the Institutional Review Board of Asan Medical Center, Republic of Korea (2016–0935). The patients/participants provided their written informed consent to participate in this study.

## Author Contributions

Conception and design: HL and ML. Development of the methodology: SS, J-HS, and ML. Data acquisition: J-HS, SS, IS, and Y-AK. Data analysis: SS, ML, and J-HS. Writing, review, and/or revision of the manuscript: ML, J-HS, SS, SK, GG, JK, and HL. All authors contributed to the article and approved the submitted version.

## Funding

This study was supported by the Asan Institute for Life Sciences, Asan Medical Center, Seoul, Korea (2018IL0733, 2019IL0733, and 2020IL0019).

## Conflict of Interest

Authors SS and Y-AK were employed by company NeogenTC Corp.

The remaining authors declare that the research was conducted in the absence of any commercial or financial relationships that could be construed as a potential conflict of interest.

## Publisher’s Note

All claims expressed in this article are solely those of the authors and do not necessarily represent those of their affiliated organizations, or those of the publisher, the editors and the reviewers. Any product that may be evaluated in this article, or claim that may be made by its manufacturer, is not guaranteed or endorsed by the publisher.

## Abbreviations

HLA, Human leukocyte antigen; MHC, Major histocompatibility complex; NGS, Next-generation sequencing; PCR, Polymerase chain reaction; TCR, T-cell receptors; WES, Whole-exome sequencing; WGS, Whole-genome sequencing.
